# Effect of Low-Frequency Repetitive Transcranial Magnetic Stimulation on Executive Function and Its Neural Mechanism: An Event-Related Potential Study

**DOI:** 10.3389/fnins.2021.701560

**Published:** 2021-10-27

**Authors:** Sishi Liu, Xianglong Wang, Junqin Ma, Kangling Wang, Zhengtao Wang, Jie Li, Jiali Chen, Hongrui Zhan, Wen Wu

**Affiliations:** ^1^Department of Rehabilitation, Zhujiang Hospital, Southern Medical University, Guangzhou, China; ^2^Rehabilitation Medical School, Southern Medical University, Guangzhou, China; ^3^Department of Rehabilitation, The Fifth Affiliated Hospital of Sun Yat-sen University, Zhuhai, China

**Keywords:** rTMS, executive function, DLPFC, P3, task switching, ERP

## Abstract

**Objective:** Executive function refers to the conscious control of thinking and behavior in psychological process. Executive dysfunction widely exists in a variety of neuropsychiatric diseases, and is closely related to the decline of daily living ability and function. This study intends to explore the effect of low-frequency repetitive transcranial magnetic stimulation (rTMS) on executive function and its neural mechanism by using event-related potential (ERP), so as to provide basis for further study on the relationship between cerebral cortex and executive function.

**Methods:** Task switching paradigm was used to study the cognitive flexibility in executive function. Thirty-one healthy subjects were randomly assigned to receive rTMS stimulations (1 Hz rTMS or sham rTMS) to the left dorsolateral prefrontal cortex (DLPFC) twice. The switching task and the electroencephalography EEG recordings were performed before (pre-rTMS/pre-sham rTMS) and immediately after the end of the rTMS application (post-rTMS/post-sham rTMS).

**Results:** The analysis of RTs showed that the main effects of switching and time were statistically significant. Further analysis revealed that the RT of rTMS stimulation was longer than sham rTMS at post-stimulation. ERP analysis showed that there was a significant switching effect in frontal and central scalp location, and the P2 amplitude in switch trials was greater than that in non-switch trials. At post-stimulation, the N2 amplitude of rTMS is more negative than that of sham rTMS at non-switch trials, whereas no such difference was found at switch trials. The P3 amplitude and LPC amplitude are significantly reduced by rTMS at post-stimulation.

**Conclusion:** Low-frequency rTMS of the left DLPFC can cause decline of cognitive flexibility in executive function, resulting in the change of N2 amplitude and the decrease of P3 and LPC components during task switching, which is of positive significance for the evaluation and treatment of executive function.

## Introduction

Executive function refers to conscious control related to thinking and behavior in psychological process, including decision-making, planning, cognitive flexibility, attention, working memory, and other cognitive processes (Guo et al., 2017; Ozga et al., 2018). These important thinking abilities can help people adapt to the complex and changeable environment. When the executive function is impaired, patients cannot make plans and cannot adjust themselves according to the rules, which is a great obstacle for patients to return to society. Although the concept of executive function was first discovered and perfected in the study of frontal lobe syndrome (DeRight, 2019), it has been recognized that executive function involves the precise network of frontal cortex and other brain regions, including parietal cortex, basal ganglia, and colliculus. A previous study (Niendam et al., 2012) has shown that the frontal cingulate parietal subcortical cognitive control network supports a wide range of executive functions. Executive dysfunction is caused by the damage of white matter connection or neurotransmitter system in related brain regions (Rabinovici et al., 2015). Therefore, executive dysfunction widely exists in a variety of neurological, mental, and systemic diseases, and is closely related to the decline of daily living ability and function.

It is well known that the DLPFC plays an important role in various higher-order cognitive functions, such as executive control, planning, working memory, and so on. In elderly subjects, the regulatory effect of DLPFC-SAI (short-latency afferent inhibition) paradigm on N100 was related to the experimental executive function (Noda et al., 2017), and in schizophrenia, the decrease of N100 modulation of TMS-evoked potentials (TEPS) by DLPFC was significantly correlated with executive function (Noda et al., 2018). These studies indicate that DLPFC plays an important role in executive function, and executive function shows asymmetry in left and right DLPFC, showing obvious left hemisphere dominance. A study Ko et al. (2008) showed that continuous theta pulse stimulation (cTBS) was applied to left and right DLPFC, compared with cTBS at the vertex (control). Only cTBS of the left DLPFC impaired Montreal card sorting task (MCST) performance and striatal dopamine neurotransmission.

Cognitive flexibility is one of the core components of executive function and plays an important role in executive control. Task switching program is usually used to study cognitive flexibility, which requires participants to switch between two tasks with different rules (Vanderhasselt et al., 2006). When participants switch between tasks, they shift their attention between one task and another, and activate a new task set in working memory. This process is usually accompanied by an increase in reaction time (RT) and error rate (ER) (Bahlmann et al., 2015). Switching cost represents the performance difference between repeated tasks and switching tasks (Strobach et al., 2018). The behavioral research of task switching paradigm (Swainson et al., 2019) is very mature, but the research on its electrophysiology is limited.

TMS is a non-invasive, safe, and reliable method for cortical (and peripheral) stimulation (He et al., 2020). Because of its painless, non-invasive physical characteristics, it can achieve virtual damage of brain regions to explore brain function and advanced cognitive function. It enables researchers to infer the causal relationship between cortical function and potential cognitive and behavioral processes, while avoiding inconsistencies in the location, volume, and nature of brain damage in clinical models (Lowe and Hall, 2018). However, the physiological mechanism of rTMS induced action is not clear. At present, some studies have confirmed that stimulation of local blood flow and metabolism (Lin et al., 2018), upregulation of brain-derived nerve growth factor, improvement of synaptic plasticity, or change of cortical excitability may be the effective mechanisms of rTMS. So far, there are few studies on the mechanism of rTMS affecting executive function by using ERP.

Event-related potential is a technology with high temporal resolution, reaching the millisecond level, which can record, analyze, and characterize the dynamic electrophysiological activities of living brain (Raz et al., 2016). ERP makes up for the low time resolution of PET and fMRI methods, and has important value in the study of the relationship between cognitive function and neural process. As for task switching, three main ERP components are particularly relevant: P200, N200, and P300 (Massa et al., 2020). P200 is a positive waveform, which reaches its peak about 200 ms after stimulation, and its amplitude is the largest at the frontal electrode. In the paradigm of task switching, some literatures show that P200 is the first component to distinguish switching and non-switching trials. The second useful component of execution process is N200, which is a negative waveform and reaches its peak between 200 and 350 ms after stimulation. N200 is related to attention system and cognitive control, reflecting the cognition of conflict or suppression of dominant responses (Ruberry et al., 2017). P300 is a typical positive waveform in the time window of 250–800 ms after stimulation. P300 components are generated in the neural network composed of frontal lobe, anterior cingulate cortex, inferior temporal lobe, and parietal cortex. The distribution of P3b in parietal lobe is related to working memory and task cognitive resource allocation, and the decrease of P3b is related to lower task performance (Hawkes et al., 2014).

In this study, we intend to investigate the effects of low-frequency rTMS on the left DLPFC to explore the effect on executive function and its neural mechanism by using ERP. Through the operation of complex task switching paradigm, the ERP components related to executive function of midline frontal, central, and parietal channels were analyzed to provide basis for further study on the relationship between cerebral cortex and executive function. Assuming that the low-frequency rTMS on the left DLPFC could cause decline of executive function during task switching, we expected an increase of RTs and decline of accuracy following rTMS as compared to sham rTMS.

## Experimental Procedures

### Participants

Thirty-one college students (mean age 23.84 ± 0.344 years old, 12 males and 19 females) were recruited. The subjects were healthy and right-handed with normal or corrected-to-normal vision. Exclusion criteria are as follows: people with metal or electronic device implantation, such as cochlear implant, pulse generator, and medical pump; color blindness or color weakness; organic or functional nervous system diseases; history of taking antipsychotics and drug abuse; and having contact with similar related experimenters. Before the experiment, subjects gave their informed consent. They were able to complete the tests intensively and conscientiously. All procedures complied with guidelines as described in the Declaration of Helsinki. The experiment was approved by the Ethical Committee of the Zhujang Hospital of Southern Medical University. The data from two females and one male were excluded from the analyses due to excessive electroencephalography EEG artifacts and baseline drifts that were difficult to correct. The remaining 28 participants (17 females, 23.89 ± 1.99 years old) were included in the analyses.

### Switching Task

The task was performed with E-prime 3.0 software (Psychology Software Tools Inc., Pittsburgh, United States), using numbers between 1 and 9 (except 5) as stimulus. The stimuli were presented on a 21-inch CRT monitor (60-Hz refresh rate), with a white background at a distance of approximately 100 cm from the participant. The task included 216 trials; in half of the trials, the numbers were shown in black and the other half in blue. In each experiment, in the center of the computer screen, the fixation was presented for 1,000 ms, a single black or blue number was presented for 500 ms, and then a blank screen was presented for 2,000 ms. Participants switch between tasks based on the color of the number. When the screen shows black numbers, judge the size of the number, press the “Q” key when it is less than 5, and press the “P” key if it is larger than 5. When the blue number is displayed, judge whether the number is odd or even. Press “Q” for odd number and “P” for even number. Instruct participants to answer as quickly and accurately as possible. Participants pressed the button with their left or right index finger and response mapping was counter-balanced across them. The number presentation is random, and the number of switch and non-switch (repeated) trials is the same. The color of the number is the same as the previous experiment, which is a repeated experiment, while the color of the number is different from the previous experiment, which is a switch experiment. Record the RT and accuracy of switch and repeated test. There is a short exercise before the experiment, and the correct rate of reaction needs to reach 80% to enter the formal test.

### Transcranial Magnetic Stimulation Parameters

rTMS pulses were delivered using a YRD CCY-I TMS stimulator (YRD, Wuhan, China) with a figure-of-eight focal coil (external diameter of each loop, 9 cm), which produced a maximum stimulator output (MSO) of 3.0 T. The subjects relaxed naturally and sat in a comfortable armchair. Stimulation was applied over the hand representation within primary motor cortex, and the EMG recording electrode was placed in the abdomen of the abductor pollicis brevis (APB) to record the motor-evoked potentials (MEPs). In the resting state, we localized the thumb area of the left motor cortex by eliciting a robust MEP, and then gradually decreased the output intensity to stimulate until the motor threshold (MT) is found, so that at least 5 out of 10 consecutive stimuli can trigger the right APB motion. The intensity of stimulation used for different subjects ranged between 44 and 75% (mean ± SD, 58.93 ± 8.42%) of maximal stimulator output with wearing EEG cap. TMS was then applied 20 min stimulus (1,040 impulses) at a frequency of 1 Hz and an amplitude of 90% of the MT at a distance of 5 cm anterior to the located left primary motor cortex. For 1 Hz stimulation, the stimulating coil was held tangentially to the skull with the coil handle pointing backward and laterally 45°away from the anterior–posterior axis, while for sham stimulation, the coil was placed vertically (at a 90° angle) to the scalp.

### Procedure

The experiment was designed as a single-blind crossover design. The subjects sat in a comfortable chair and received 20 min of treatment with 1 Hz rTMS or sham rTMS on the left DLPFC. In each session, the switching task and the EEG recordings were performed before (pre-rTMS/pre-sham rTMS) and immediately after the end of the rTMS application (post-rTMS/post-sham rTMS), which lasted approximately 12 min, respectively. All subjects were wearing the 32-channel EEG cap during the whole session (Levit-Binnun et al., 2010). The interval between rTMS stimulation and task should be as short as possible (interval range 7–11 min). Each subject received two experiments (including 1 Hz rTMS and sham rTMS stimulation) with an average interval of 1 week to eliminate possible carry-over effects. The sequence of low-frequency and sham rTMS stimuli was balanced among participants to minimize possible sequence effects.

### Electroencephalography Recording and Data Acquisition

The incorrect response trials were excluded from analysis. EEE was recorded with the 64-channel BIOSEMI Active Two system, which used an electrode cap with 32 Ag/AgCl electrodes mounted according to the international 10–20 system. We used the average value of bilateral mastoid as the reference when recoding EEG online. The sampling rate of EEG was 2,048 Hz, and the bandpass filtered from 0.1 to 100 Hz. The electrode impedance was kept below 5 kΩ.

We used EEGLAB (version 13_0_0b) for offline analysis of EEG data. EEGLAB is a MATLAB (R2013b, MathWorks, Natick, MA, United States) open source toolbox. Data were bandpass filtered at 0.1–50 Hz while notch filtering (49–51 Hz). Change the sampling rate to 500 Hz. EEG recordings were segmented into epochs from -100 to 800 ms relative to the onset of stimulus. A baseline correction (pre-stimulus interval) and automatic artifact rejection (±100 μV) were executed. Remove EOG and EMG activities using independent component analysis (ICA). We observed ERP waveforms and found that the basic characteristics of the ERP curve in the frontal channels (F3, Fz, F4) were consistent, as were the three electrodes in the central channels (C3, Cz, C4) and the parietal channels (P3, Pz, P4). According to previous studies (Küper et al., 2017; Pestalozzi et al., 2020) and the characteristics of this experiment, the average amplitudes of P2 (140–240 ms), N2 (260–340 ms), P3 (360–450 ms), and late components (500–800 ms) were measured across the three brain regions, including frontal (F3, Fz, F4), central (C3, Cz, C4), and parietal (P3, Pz, P4) electrodes.

### Data Analysis

SPSS Statistics 22 (IBM Corp, Armonk, NY, United States) was implemented for statistical analysis. The statistical threshold was set at *p* < 0.05.

Data corresponding to correct responses were analyzed. Repeated measurement ANOVA of 2 (stimulation factors: low-frequency rTMS, sham rTMS) × 2 (time factors: pre-stimulation, post-stimulation) × 2 (switching factors: switch, non-switch) was performed with response time and accuracy as dependent variables.

Multiple channel ERP data were analyzed by repeated measurement ANOVA of 2 (stimulation factors: low-frequency rTMS, sham rTMS) × 2 (time factors: pre-stimulation, post-stimulation) × 2 (switching factors: switch, non-switch). Significant ANOVA effects were further analyzed with pairwise *t*-test comparisons.

## Results

The subjects did not report side effects during or after the experiment. All data were checked the Q-Q plots to meet the assumption of normality.

### Behavioral Data

**FIGURE 1:**
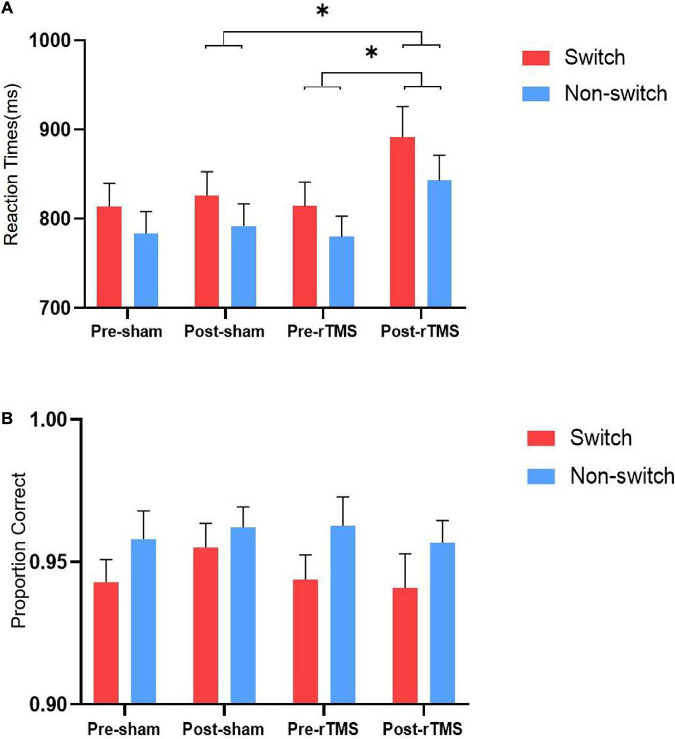
Reaction times **(A)** and accuracy rates **(B)** on switch and non-switch conditions for different stimulation conditions. ^∗^ Significant difference between stimulation conditions, *p* < 0.05. Error bars represent SEM.

Results of RTs and accuracy rates on switch and non-switch conditions for different stimulation conditions are presented in Figure 1.

The results of ANOVA of response time showed that the main effects of switching [*F*_(__1,__27)_ = 58.819, *p* < 0.001] and time [*F*_(__1,__27)_ = 9.729, *p* = 0.004] were statistically significant. The main effect of stimulation was not significant [*F*_(__1,__27)_ = 2.169, *p* = 0.152]. The interaction “stimulation” × “time” was significant [*F*_(__1,__27)_ = 5.084, *p* = 0.032]. Further analysis revealed that the RT of rTMS stimulation was longer than sham rTMS at post-stimulation [*F*_(__1,__27)_ = 4.192, *p* = 0.05], whereas no significant difference was found at pre-stimulation [*F*_(__1,__27)_ = 0.009, *p* = 0.926]. The interactions stimulation × switching type, time × switching type, and stimulation × time × switching type were not significant [*F*_(__1,__27)_ = 1.408, *p* = 0.246; *F*_(__1,__27)_ = 3.87, *p* = 0.06; and *F*_(__1,__27)_ = 1.318, *p* = 0.261, respectively].

The results of ANOVA of the accuracy showed that the main effect of switching [*F*_(__1,__27)_ = 10.403, *p* = 0.003] was significant, whereas stimulation [*F*_(__1,__27)_ = 0.623, *p* = 0.437] and time [*F*_(__1,__27)_ = 0.101, *p* = 0.753] were not significant. The interactions stimulation × time, stimulation × switching, time × switching type, and stimulation × time × switching type were not significant [*F*_(__1,__27)_ = 2.448, *p* = 0.129; *F*_(__1,__27)_ = 1.513, *p* = 0.229; *F*_(__1,__27)_ = 0.612, *p* = 0.441; and *F*_(__1,__27)_ = 0.275, *p* = 0.604, respectively].

### Event-Related Potential Data

**FIGURE 2:**
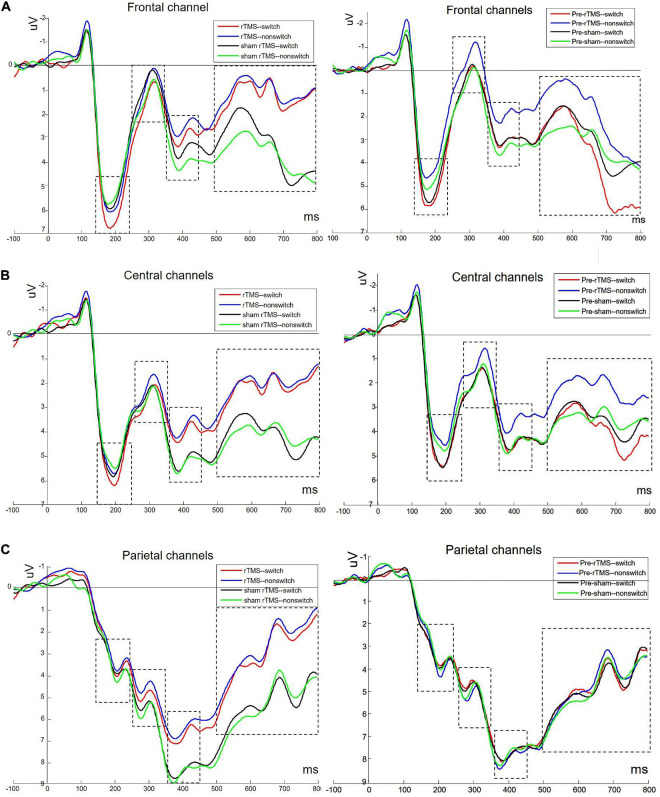
Grand averaged ERPs to switch and non-switch conditions at frontal (**A**: average of channels 4, 27, and 31), central (**B**: average of channels 8, 23, and 32), and parietal channels (**C**: average of channels 12, 13, and 19) for 1 Hz rTMS and sham rTMS stimulations, including post-stimulation and pre-stimulation.

**FIGURE 3:**
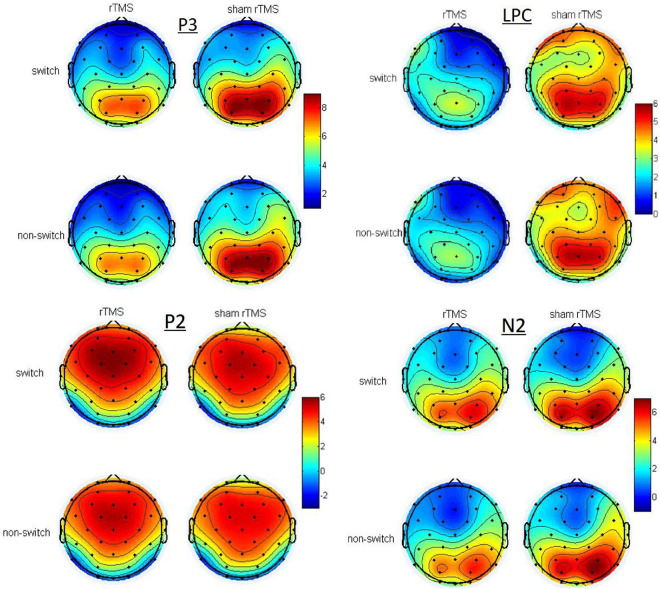
Topographic maps of the voltage distribution of P2, N2, P3, and LPC to switch and non-switch conditions for 1 Hz rTMS and sham rTMS stimulations at post-stimulation.

Figure 2 depicts the grand averaged ERPs to switch and non-switch conditions at frontal, central, and parietal channels for 1 Hz rTMS and sham rTMS stimulations, including post-stimulation and pre-stimulation. Figure 3 presents voltage distribution of P2, N2, P3, and LPC to switch and non-switch conditions for 1 Hz rTMS and sham rTMS stimulations.

#### P2 (140–240 ms Post-stimulus)

Analysis of the P2 component in the frontal channels revealed a significant main effect of time [*F*_(__1,__27)_ = 5.481, *p* = 0.027], indicating a larger P2 at post-stimulation (5.074 ± 0.573 μV) than at pre-stimulation (4.31 ± 0.604 μV). The main effect of switching type was also significant [*F*_(__1,__27)_ = 4.671, *p* = 0.04]. The amplitude of the switch trials (4.989 ± 0.56 μV) was greater than the non-switch trials (4.395 ± 0.604 μV). No other main effect or interaction effects were found [*F*_(__1,__27)_ = 0.531, *p* = 0.472; stimulation × time *F*_(__1,__27)_ = 0.826, *p* = 0.371; stimulation × switching *F*_(__1,__27)_ = 1.735, *p* = 0.199; time × switching *F*_(__1,__27)_ = 0.749, *p* = 0.395; and stimulation × time × switching *F*_(__1,__27)_ = 0.103, *p* = 0.751, respectively].

Analysis of the P2 component in the central channels revealed a marginally significant main effect of time [*F*_(__1,__27)_ = 4.075, *p* = 0.054], indicating a larger P2 at post-stimulation (4.779 ± 0.519 μV) than at pre-stimulation (4.148 ± 0.519 μV). The main effect of switching type was also significant [*F*_(__1,__27)_ = 7.196, *p* = 0.012]. The amplitude of the switch trials (4.736 ± 0.488 μV) was greater than the non-switch trials (4.191 ± 0.522 μV). No other main effect or interaction effects were found [*F*_(__1,__27)_ = 0.087, *p* = 0.771; stimulation × time *F*_(__1,__27)_ = 0.151, *p* = 0.701; stimulation × switching *F*_(__1,__27)_ = 0.712, *p* = 0.406; time × switching *F*_(__1,__27)_ = 1.022, *p* = 0.321; and stimulation × time × switching *F*_(__1,__27)_ = 0.129, *p* = 0.722, respectively]. There were no main effects or interactions in the parietal channels.

#### N2 (260–340 ms Post-stimulus)

Analysis of the N2 in the frontal channels revealed a marginally significant main effect of time [*F*_(__1,__27)_ = 2.992, *p* = 0.095]. There was a marginally significant interaction between stimulation × switching [*F*_(__1,__27)_ = 3.954, *p* = 0.057]. Through further analysis, we found a significant main effect of switching type [*t*(27) = –2.199, *p* = 0.037] with sham rTMS, whereas no such difference was found within rTMS stimulation [*t*(27) = 1.109, *p* = 0.277]. The N2 amplitude of switch trials (0.533 ± 0.628 μV) was more pronounced (more negative) than non-switch trials (1.031 ± 0.544 μV) with sham rTMS. The main effects of stimulation [*F*_(__1,__27)_ = 0.084, *p* = 0.774] and switching [*F*_(__1,__27)_ = 0.119, *p* = 0.733] were not significant. The interactions stimulation × time, time × switching, and stimulation × time × switching [*F*_(__1,__27)_ = 0.408, *p* = 0.528; *F*_(__1,__27)_ = 0.053, *p* = 0.819; *F*_(__1,__27)_ = 0.031, *p* = 0.862] were not significant.

Analysis of the N2 in the central channels revealed a marginally significant main effect of time [*F*_(__1,__27)_ = 3.121, *p* = 0.089]. The main effects of stimulation [*F*_(__1,__27)_ = 0.268, *p* = 0.609] and switching [*F*_(__1,__27)_ = 0.924, *p* = 0.345] were not significant. The interactions stimulation × time, stimulation × switching, time × switching, and stimulation × time × switching were not significant [*F*_(__1,__27)_ = 0.007, *p* = 0.936; *F*_(__1,__27)_ = 2.176, *p* = 0.152; *F*_(__1,__27)_ = 0.176, *p* = 0.678; *F*_(__1,__27)_ = 0.025, *p* = 0.876, respectively].

Analysis of the N2 in the parietal channels revealed a three-way interaction between stimulation × time × switching [*F*_(__1,__27)_ = 4.702, *p* = 0.039]. At non-switch trials, further analysis revealed that there was a significant main effect of stimulation [*F*_(__1,__27)_ = 4.62, *p* = 0.041]. The interaction between the stimulation × time was also significant [*F*_(__1,__27)_ = 11.974, *p* = 0.002]. Further analysis revealed a significant main effect of stimulation [*t*(27) = 3.08, *p* = 0.005] at post-stimulation, whereas no such difference was found at pre-stimulation [*t*(27) = –0.965, *p* = 0.343]. The N2 amplitude of rTMS (4.615 ± 0.681 μV) was more pronounced (more negative) than sham rTMS (6.006 ± 0.55 μV) at post-stimulation. There were no main effects or interaction effects at switch trials.

#### P3 (360–450 ms Post-stimulus)

There were no main effects or interactions in the frontal and central channels. Analysis of the P3 in the parietal channels revealed a significant main effect of stimulation [*F*_(__1,__27)_ = 7.876, *p* = 0.009], which revealed significant decreased P3 amplitude in rTMS (7.128 ± 0.588 μV) in contrast to sham rTMS (8.091 ± 0.694 μV). There was a significant interaction between stimulation × time [*F*_(__1,__27)_ = 6.736, *p* = 0.015]. Through further analysis, we found a significant main effect of stimulation [*t*(27) = 2.84, *p* = 0.008] at post-stimulation, whereas no such difference was found at pre-stimulation [*t*(27) = 0.562, *p* = 0.579]. There was a significant decreased P3 amplitude in rTMS (6.665 ± 0.649 μV) in contrast to sham rTMS (8.474 ± 0.707 μV) at post-stimulation. The main effects of time [*F*_(__1,__27)_ = 0.032, *p* = 0.86] and switching [*F*_(__1,__27)_ < 0.001, *p* = 0.985] were not significant. The interactions stimulation × switching, time × switching, and stimulation × time × switching [*F*_(__1,__27)_ = 0.822, *p* = 0.373; *F*_(__1,__27)_ = 0.793, *p* = 0.381; *F*_(__1,__27)_ = 1.601, *p* = 0.217, respectively] were not significant.

#### Late Positive Component (LPC, 500–800 ms Post-stimulus)

Analysis of the LPC in the frontal channels revealed a significant main effect of stimulation [*F*_(__1,__27)_ = 7.019, *p* = 0.013], which revealed significant decreased LPC amplitude in rTMS (1.999 ± 0.503 μV) in contrast to sham rTMS (3.316 ± 0.736 μV). There was a marginally significant interaction between stimulation × time [*F*_(__1,__27)_ = 4.136, *p* = 0.052]. Through further analysis, we found a significant main effect of stimulation [*t*(27) = 2.631, *p* = 0.014] at post-stimulation, whereas no such difference was found at pre-stimulation [*t*(27) = 0.648, *p* = 0.522]. There was a significant decreased LPC amplitude in rTMS (1.205 ± 0.643 μV) in contrast to sham rTMS (3.543 ± 0.736 μV) at post-stimulation. No other main effects or interaction effects were found.

Analysis of the LPC in the central channels revealed a significant main effect of stimulation [*F*_(__1,__27)_ = 7.436, *p* = 0.011], which revealed significant decreased LPC amplitude in rTMS (2.51 ± 0.398 μV) in contrast to sham rTMS (3.829 ± 0.622 μV). No other main effects or interaction effects were found.

Analysis of the LPC in the parietal channels revealed a significant main effect of stimulation [*F*_(__1,__27)_ = 14.767, *p* = 0.001], which revealed significant decreased LPC amplitude in rTMS (3.731 ± 0.449 μV) in contrast to sham rTMS (5.079 ± 0.667 μV). There was a significant interaction between stimulation × time [*F*_(__1,__27)_ = 9.804, *p* = 0.004]. Through further analysis, we found a significant main effect of stimulation [*t*(27) = 3.742, *p* = 0.001] at post-stimulation, whereas no such difference was found at pre-stimulation [*t*(27) = 0.848, *p* = 0.404]. There was a significant decreased LPC amplitude in rTMS (2.93 ± 0.443 μV) in contrast to sham rTMS (5.404 ± 0.714 μV) at post-stimulation. No other main effects or interaction effects were found.

## Discussion

We investigated behavioral and ERP results of executive function in healthy subjects with rTMS stimulation compared with sham rTMS. At the behavioral level, we found poorer performance of subjects with rTMS stimulation, resulting in longer RTs. The average response time of switch trials was significantly longer than that of non-switch trials. On an electrophysiological level, there is an obvious switching effect of P2 amplitude in frontal and central channels. At post-stimulation, the N2 amplitude of rTMS is more negative than that of sham rTMS at non-switch trials, whereas no such difference was found at switch trials. The P3 amplitude and LPC amplitude are significantly reduced by rTMS at post-stimulation, whereas no such difference was found at pre-stimulation. These results indicate that rTMS of the left DLPFC resulted in significant impairments in the task switching of executive function.

Many studies have found that the change of executive function is closely related to the changes of ERP components, which shows the substantial change of event-related brain electrical activities. For example, in the study of the executive function of dyslexic adolescents, it was found that (Horowitz-Kraus, 2012) in the implementation task of the Wisconsin Card Sorting Test (WCST), ERP differences were found under the condition of “target-locked,” and the ERP components (N100, P300) of dyslexics were lower than those of skilled readers.

In this study, for the P2 component, it is found that there is a significant switching effect in frontal and central scalp locations, and the P2 amplitude in switch trials was greater than that in non-switch trials. This shows that the impact of task switching on P2 component reflects the recruitment of processes related to the switch of task sets, which is consistent with other research results (Raz et al., 2016). An additional active reconfiguration process is supposed to be needed in switch trials in order to activate the task set of the current trial and to inhibit the task set of the previous trial (Fiedler et al., 2009). Higher cognitive control resources, reflected by larger P2 amplitude, are needed during switch trials (Massa et al., 2020). P2 is believed to originate from the visual association cortex and is related to the task relevance of evaluating stimuli. A study (Choi et al., 2014) has found that the increase of P200 amplitude of auditory oddball task after 3 weeks of rTMS treatment is related to the improvement of depression symptoms in drug-resistant depression patients, which may play a role by suppressing irrelevant features (negative stimuli) or enhancing related features (positive stimuli). In this study, rTMS stimulation had no effect on the switch effect of P2 components, indicating that rTMS stimulation of the left DLPFC had little effect on the early perception stage of task switching in healthy people.

In the parietal scalp locations, at post-stimulation, the N2 amplitude of rTMS is more negative than that of sham rTMS at non-switch trials, whereas no such difference was found at switch trials. N2 is considered to reflect the successful inhibition control, and its neural sources include frontal lobe and superior temporal cortex, as well as anterior cingulate cortex. The frontal cortex is a key area for sensory information integration, and also a key area for controlling and allocating attention resources (Nathou et al., 2018). Some results suggested that the difference between switch and repeat trials is due primarily to differences in the strength of responses within a statistically indistinguishable frontoparietal brain network, which indicated that the activity on switch trials is not qualitatively different from that on repeat trials (Wylie et al., 2009). The difference between switch and non-switch N2 suggests that during implementation of task sets (i.e., stimulus–response sets), interference from the currently irrelevant S–R set has to be overcome (inhibited) (Gajewski et al., 2018). However, the switch-N2 effect was not found in this study, which is consistent with another relevant study (Zhuo et al., 2021). The current study found that N2 was less sensitive to task switching, but more sensitive to rTMS stimulation. The N2 amplitude of rTMS stimulation was more negative than that of sham rTMS for non-switch trials at post-stimulation, which indicated that inhibition control of rTMS stimulation was more effective for non-switch trials. The low-frequency rTMS stimulation inhibited the cortical excitability, changing the N2 amplitude of task switching, which may reflect the active “top-down” suppression of dominant response.

Analysis of the P3 in the parietal channels showed a significant effect of rTMS stimulation at post-stimulation, whereas no such difference was found at pre-stimulation. The amplitude of P3 induced by low-frequency rTMS stimulation was smaller than that induced by sham stimulation. The study gave evidence that prefrontal areas of the left hemisphere play a major role in eliciting the P3 component (Evers et al., 2001). It seems to be a consensus that P3 has become a neurophysiological indicator for information processing and updating in working memory (Gu et al., 2019). In many studies, P3 amplitude is associated with the success of task performance, including attention and memory (Downes et al., 2017). Studies have found that the P3 amplitude was larger for those trials that responded faster (Jost et al., 2008). The amplitude of P300, MMN, and N400 increased after 10 Hz high-frequency rTMS stimulation in schizophrenic patients (Lin et al., 2018), which can be used as a valuable electrophysiological reference for evaluating the therapeutic effect of rTMS in schizophrenia. In this study, low-frequency rTMS stimulation of the left DLPFC reduced the amplitude of P3, which may damage the information processing and updating process in task switching, and this is consistent with our behavioral results of impaired performance. There were significant changes of P3 amplitude stimulated by rTMS in the parietal areas far away from the stimulation, which showed that rTMS could affect the function of the surrounding cortex in relatively distant areas (Nathou et al., 2018), and the affected areas were related to the functional state of the brain, which was also confirmed in the relevant fMRI experiment (van den Heuvel et al., 2013).

Analysis of the LPC in frontal and parietal channels showed that there was a significant effect of rTMS stimulation at post-stimulation, whereas no such difference was found at pre-stimulation. The amplitude of low-frequency rTMS stimulation was smaller than that of sham stimulation. In the previous task switching paradigm study, healthy controls induced a more positive posterior switch positivity (PSP) (Elchlepp et al., 2012) waveform under the switch cues than the non-switch cues, while the amyotrophic lateral sclerosis (ALS) patients lacked switch-related ERP modulations due to executive dysfunction (Lange et al., 2016). The reason why there is no PSP waveform in the results of this study may be the different switching paradigms used. In this study, there was no cue before the stimulus was presented, but the switch or non-switch stimulus was directly presented. Therefore, there was no process of identifying clues in advance and preparing for anticipation, and there were differences in cognitive processing. This component of time window of 500–800 ms is usually called the LPC, which is found to be related to the cognitive process of conflict resolution and self-monitoring in speech production in language switching study (Pestalozzi et al., 2020). The LPC was decreased in frontal and parietal channels, suggesting the involvement of the frontoparietal network in task-switch processes. The LPC may reflect the activity of a neural mechanism that supports processes of the task set reconfiguration involved in the task-switch processes (Bisiacchi et al., 2009). Low-frequency rTMS stimulation of the left DLPFC reduces the amplitude of LPC, which may indicate that the task set reconfiguration efficiency in the process of task execution is affected and the frontal lobe’s recruitment of cognitive resources is reduced.

In this study, the effect of low-frequency rTMS on behavior results is significant, which is consistent with some research results (Lowe et al., 2018). As there was no significant stimulation × time × switching interaction in behavior, it seems that rTMS did not cause behavioral changes in switching tasks; however, the significant interaction was found in ERP results, which indicates that the change of brain activity in these executive control areas following stimulation seems to be related to task switching. In healthy bilinguals who have a high level of attentional control, the effects of prefrontal cTBS stimulation on language control showed no behavioral changes, but ERP changes (Pestalozzi et al., 2020), which were different from patients with lesions in the DLPFC. Many studies have shown that ERP measurement may be more sensitive to the impact of rTMS than behavioral data. Gottesman and Gould (2003) and Grossheinrich et al. (2013) introduced the concept of endophenotype, that is, “measurable components that are invisible to the naked eye along the pathway between disease and distal genotype.” Compared with behavioral measures such as RT and accuracy, ERP measurement is an invisible component. The concept of endophenotype explains the interaction change in endogenous cognitive ERP components without behavioral changes.

There are still some deficiencies in this study. Firstly, the limitations of the sham rTMS approach used in this paper do exist. In recent years, new progress has been made in the study of TMS, and the research on sham TMS approaches has become more and more in-depth. Turning the coil on its side is not really and effective sham condition. It is rather difficult to determine whether or not there is residual brain stimulation in such cases (Duecker and Sack, 2015). Therefore, more appropriate TMS control conditions should be used in the future. Secondly, executive function includes multiple sub-components, including inhibitory control, working memory, and decision-making, but this study only studied one of them, cognitive flexibility through task switching. In the future, we plan to study other sub-components of executive function.

## Conclusion

In conclusion, we present a novel investigation of cognitive function and associated brain mechanisms and dynamics in rTMS stimulation. Our results clearly indicate impairments in executive function in the rTMS condition, accompanied by significant alterations in neural activation. Low-frequency rTMS of the left DLPFC can cause decline of executive function, resulting in the change of N2 amplitude and the decrease of amplitude of P3 component and LPC component during the performance of task switching. Low-frequency rTMS has an effect on many stages of cognitive time course of executive function in healthy subjects. The influence of early perception stage is not significant, and the effect is obvious in parietal area, which has positive significance for the evaluation and treatment of executive function.

## Data Availability Statement

The raw data supporting the conclusions of this article will be made available by the authors, without undue reservation.

## Ethics Statement

The studies involving human participants were reviewed and approved by the Ethical Committee of the Zhujang Hospital of Southern Medical University. The patients/participants provided their written informed consent to participate in this study.

## Author Contributions

XW contributed to the experimental operation and data processing. SL, JM, KW, ZW, JL, JC, HZ, and WW contributed to the experimental design. WW was helpful in the whole process of the experiment and the writing of the manuscript. All authors contributed to the article and approved the submitted version.

## Conflict of Interest

The authors declare that the research was conducted in the absence of any commercial or financial relationships that could be construed as a potential conflict of interest.

## Publisher’s Note

All claims expressed in this article are solely those of the authors and do not necessarily represent those of their affiliated organizations, or those of the publisher, the editors and the reviewers. Any product that may be evaluated in this article, or claim that may be made by its manufacturer, is not guaranteed or endorsed by the publisher.
